# Surface Integrity of Pure AW-1370 and TiC-Reinforced Aluminum WAAM Wires Under Unidirectional Sliding Contact

**DOI:** 10.3390/ma19091898

**Published:** 2026-05-05

**Authors:** Nuria Cuadrado, Giselle Ramirez, Alejandra Torres, J. Antonio Travieso-Rodriguez, Jordi Llumà, Geir Kvam-Langelandsvik, Ida Westermann, Montserrat Vilaseca

**Affiliations:** 1 Eurecat, Centre Tecnològic de Catalunya, Unit of Metallic and Ceramic Materials, Plaça de la Ciència 2, 08243 Manresa, Spain; 2 MECAMAT, Facultat de Ciències i Enginyeries, Universitat de Vic—Universitat Central de Catalunya, C. de la Laura, 13, 08500 Vic, Spain; 3 Group of CIEFMA, Department of Materials Science and Engineering, Barcelona Research Centre in Multiscale Science and Engineering (CCEM), Universitat Politècnica de Catalunya—BarcelonaTech (UPC), Av. Eduard Maristany, 16, 08019 Barcelona, Spain; 4 Departamento de Ingeniería Mecánica, Escuela Politécnica Nacional, Quito 170517, Ecuador; 5 Department of Mechanical Engineering, Universitat Politècnica de Catalunya—BarcelonaTech (UPC), Av. Eduard Maristany 10-14, 08019 Barcelona, Spain; 6 SINTEF Industry, Materials and Nanotechnology, Richard Birkelands veg 2B, 7034 Trondheim, Norway; 7 Department of Materials Science and Engineering, NTNU Norwegian University of Science and Technology, 7491 Trondheim, Norway

**Keywords:** wear mechanisms, aluminum alloys, metal matrix composites, hardmetal tool, wire drawing

## Abstract

Wire arc additive manufacturing (WAAM) demands aluminum feedstock with tightly controlled diameter and high surface integrity. Adding hard TiC nanoparticles is a viable route to enhance the mechanical response of Al wires, yet the associated increase in contact severity can accelerate the wear of wire processing tools, particularly cemented carbide dies. This study elucidates the unidirectional sliding interaction between a TiC reinforced Al WAAM wire, and a WC/Co die material containing 5 wt% Co, using a modified scratch testing configuration under dry and lubricated conditions. Two dominant mechanisms are identified: (i) aluminum adhesion on the die surface and (ii) third body abrasion arising from WC particle pull out, promoted by preferential degradation of the cobalt binder. The presence of TiC nanoparticles reduces both the extent of Al transfer and the intensity of third body abrasion, an effect that is further amplified by lubrication. Consistently, lubrication also diminishes surface defects on the wire after sliding. The results provide a mechanistic basis to balance wire strengthening with tool life and highlight practical levers—nanoparticle reinforcement and lubrication strategies—for mitigating die damage while preserving WAAM wire surface quality.

## 1. Introduction

Wire arc additive manufacturing (WAAM) is a cutting-edge manufacturing technology that enables medium-to large component production. As a direct energy deposition (DED) process, WAAM uses an electric arc to melt a wire feedstock, which is deposited layer-by-layer via a robotic arm [1,2,3,4]. Compared to laser powder bed fusion (LPBF), WAAM offers distinct advantages, including higher deposition rates, larger build envelopes, a lower health hazard, and lower investment and production costs [1,2,3,4]. These benefits make it particularly attractive for manufacturing light-weight components from materials with high laser reflectivity such as aluminum, copper, and magnesium, which are challenging for powder-based methods [1,4,5]. Despite its potential, the widespread adoption of WAAM, especially for aluminum alloys, is hindered by several process-related challenges. The quality of the final printed components is critically dependent on the feedstock wire. It has been reported that surface irregularities and porosity in the wire can act as hydrogen reservoirs during deposition, leading to porosity in the final components and deterioration of its mechanical strength [2,4]. A limited number of studies addresses the surface finishing of wires to reduce porosity and crack growth in the final printed component [6]. Issues such as porosity, microstructure inhomogeneity, and suboptimal mechanical properties in the deposited part still need to be effectively addressed [4,5,6,7,8,9,10]. Consequently, producing WAAM feedstock with a high-density, a homogeneous diameter, and an optimal surface integrity is paramount for achieving reliable, high-performance industrial components [5,7,8].

A promising solution based on metal matrix composites (MMCs) is being explored to address the common drawbacks of WAAM. MMC consists of a metal matrix reinforced with dispersed metallic, ceramic [11] particles, engineered to tailor solidification and its microstructure [11,12,13]. The addition of nanoparticles such as TiC or TiB_2_ to the feedstock facilitates the formation of fine, equiaxial grains thorough enhanced heterogenous nucleation during solidification [4,5,12,14]. This manufacturing procedure results in reduced hot-cracking susceptibility and, therefore, the printed component strength is improved [4,5,12,14,15]. However, to ensure these enhanced wires are suitable for WAAM, the post-processing wire drawing step to achieve the wire reduction is essential. This cold deformation process pulls the wire through a sequence of dies under lubrication conditions until it reaches the desired diameter required for WAAM [16].

The wire drawing process for these new MMC materials presents specific tribological challenges. A typical die material to draw aluminum is cemented carbides, made from hard refractory metal carbides and bonding metals through a power metallurgy process. In terms of drawing operations, the most common is composed of tungsten carbide (WC) embedded in a cobalt (Co) matrix [17]. This material is well-known for its outstanding wear resistance, low coefficient of friction (COF), and superior resistance to abrasion and corrosion [17,18]. However, die wear strongly affects the dimensional tolerance, roundness and surface quality of the drawn aluminum wire (e.g., scratches and pick-up) and shortens die life. For this reason, minimizing die wear is essential [18,19] to obtain reliable aluminum wire materials for WAAM. Consequently, optimizing friction conditions during drawing is critical to achieve the target wire diameter tight tolerances and surface integrity stable WAAM feeding [20,21].

The main wear mechanism during the wire drawing process of metals such as aluminum is adhesion, which is caused by the high pressures involved in the process [20,21]. Thus, controlling and reducing the coefficient of friction (COF) is critical to preserve die life and wire surface finish. The adhered and bounded aluminum in localized areas has two main drawbacks in comparison with the original feedstock: the bounded aluminum is harder and has a higher oxygen concentration [21,22,23]. These adhered aluminum features promote a two-body abrasive wear mechanism on the wire surface [24,25] which negatively affects the surface integrity of the aluminum wire. This deterioration in surface integrity increases the likelihood of porosity and crack susceptibility. Moreover, an increase in force is needed to debond the aluminum particle from the die surface, resulting in the presence of aluminum debris (micro-plough) [22] between the die and the wire. The presence of aluminum debris initiates both a third-body wear mechanism and the initiation of crack formation. While the wear of hardmetal against conventional alloys is broadly understood, the tribological interaction between WC/Co dies and novel TiC-reinforced aluminum WAAM wires remains uncharacterized. Specifically, to understand the initial wear mechanism represents a crucial first step toward improving the surface quality of WAAM aluminum wires and extending die service life during drawing operations. This aspect is particularly important for the future design or selection of the most suitable cemented carbide or cermet, based on their microstructure and properties [17].

Considering this aspects, the main objective of this study is to understand and document the surface interaction between the reinforcement nanoparticles and the WC/Co microstructure under sliding contact conditions, specifically in terms of critical micro-damage occurring in both materials To achieve this aim, a unidirectional sliding test was applied, using WC/Co balls sliding against (1) a pure EN AW-1370 aluminum wire and (2) wire reinforced with TiC nanoparticles. Tests were conducted under dry and lubricated conditions to assess the potential for mitigating wear. The resulting wear tracks on both the wire and the hardmetal counter body were examined by scanning electron microscopy to identify surface damage and wear mechanisms at room temperature.

The novelty of this study lies in its systematic characterization of this specific tribo-system, which has not been previously studied in detail, particularly with regard to the effect of TiC nanoparticle reinforcement and lubrication on the interfacial wear response. The main contribution of the paper is the mechanistic understanding of how reinforcement nanoparticles interact with WC/Co under sliding conditions, and how this interaction influences damage development at the contact interface. These findings provide a first step toward improving the surface quality of WAAM aluminum wires and extending die service life in drawing operations.

Although the results cannot be directly applied to a drawing process, because the sliding interaction evaluated in this study does not replicate the industrial conditions, by this systematic methodology aims to analyze each variable in a controlled and isolated manner at a laboratory scale in order to better understand the interaction between the die material and nanoparticle-reinforced WAAM feedstocks for future improvements in materials design.

## 2. Materials and Methods

### 2.1. Materials

Two wire materials were investigated, commercially pure EN AW-1370 (Al-1370) and EN AW-1370 reinforced with 2 wt% of TiC nanoparticles (Al-2%TiC). These materials were selected to characterize the tribological interaction under lubricated and dry conditions between WC/Co and the wire to emulate the diewire tribosystem in wire drawing. TiC nanoparticles were introduced in Al-1370 using a novel screw extrusion method developed by Langelandsvik et al. [4,5,26,27]. This methodology enables aluminum wires with TiC nanoparticles to improve hardness and reduce porosity and crack susceptibility. The analyzed wires were reduced from a feedstock wire of 3.0 mm diameter on a drawing machine (DWU-2/S, Friedr. Krollmann, Altena, Germany) until a diameter of 2 mm was reached. The selection of this reduction is because it repreesents the last drawing pass before use and is critical for the final surface quality of the wire [26]. EN AW-1370 pellets with a purity of 99.7 wt.% (Hydro Karmøy, Håvik, Norway) were used to produce the aluminum wire feedstock. In Al-2%TiC, TiC ceramic nano powder was introduced as a reinforcement. The size of the TiC nano powder ranges from 40 nm to 60 nm (US Research Nanomaterials, Houston, TX, USA) and was introduced using the prototype screw extruder machine [12,26] located at the Department of Materials Science and Engineering of the Norwegian University of Science and Technology (NTNU).

A non-commercialmineral-based oil containing ester-based additives was selected based on the author’s previous works [12] and used in all lubricated tests. Its kinematic viscosity at 40 °C was 170 mm^2^/s, while its flash point and pour point were 215 °C and −3 °C, respectively.

### 2.2. Mechanical and Tribological Characterization

Mechanical characterization for the two analysed wires includes both tensile and hardness tests. Tensile tests were performed in the longitudinal orientation and according to the ISO 6892-1 standard. The tensile specimens were machined as specified in Annex C of ISO 6892-1, with a width of 2.0 mm and a parallel length (Lc) of 90 mm, following the methodology described in [12]. Strain measurements were directly derived from the head displacement. During the test and up to the end of the elastic deformation, a strain rate of 2.5 mm/min was maintained. A total of three specimens per material were used.

Vickers hardness microindentation HV0.5 (Micrometer HV0.5, Future Tech, FM-700, Kawasaki, Japan) tests of Al-1370 and Al-2%TiC alloys were performed at a 500-g force to obtain the hardness of the alloys. Three indentations per material were performed. The resulting mechanical properties are presented in Table 1, with each value corresponding to the average of three measurements along with their standard deviation. These values have also been reported in previous works by the authors [12].

**Figure 1:**
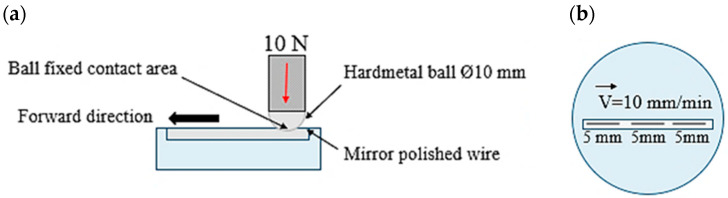
(**a**) Scheme of the ball-on-wire scratch test configuration and (**b**) wire sample diagram indicating the scratch tracks per each wire and the test direction. Tests are performed in dry and lubricated conditions. Images adpated from [12].

Tribological characterization was performed by the modified scratch test. In agreement with previous works [28], the modified scratch test consists of drawing a spherical tip across the specimen flat surface at a constant speed (10 mm/min). A WC/Co ball (Ø = 10 mm) is used, and a normal force of 10 N is applied, resulting in an approximate contact pressure of 470 MPa. This value was chosen to investigate wear mechanisms rather than to replicate typical drawing process conditions, which depend on factors such as reduction percentage, die geometry, temperature, and lubrication. Figure 1 shows a scheme of the modified scratch test used to characterize the COF [12]. Sliding mode was carried out in a unidirectional motion along 5 mm of the track distance using a Micro Hardness Tester (MHT) (CSM Instruments, Filderstadt, Germany). The chemical composition, microstructure, hardness, and surface roughness of the commercial hardmetal balls are described in Table 2.

Tests were carried out in dry and lubricated conditions at 21 ± 1 °C. Prior to testing, Al-1370 and Al-2 wt% TiC wires were polished to a mirror finish using 0.03 µm colloidal silica, ensuring a reproducible contact zone [12]. For each material and condition (dry/lubricated), three scratches were performed (*n* = 3). After each of the three scratch tests, the ball was removed and analyzed using field emission scanning electron microscopy (FE-SEM) (Ultra Zeiss, ZEISS, Dublin, CA, USA), equipped with an energy-dispersive X-ray spectrometry (EDX) OXFORD 5 detector to determine the wear mechanisms. After the first scratch, the ball was repositioned into the holder with the same initial orientation, and a second test was conducted on a fresh wire surface. After the second scratch, the WC/Co ball was again analyzed by SEM. Finally, a third scratch test was carried out following the same procedure [12]. Scratch tracks were placed at different positions along the wire samples, as shown in Figure 1b. The initial running-in period was neglected in COF evaluation, meaning that COF mean values were determined under steady-state conditions. The scratching procedure was applied to understand and document the first stages of wear mechanisms on WC/Co hardmetal, which eventually can modify the surface quality of WAAM wires. Thus, sliding contact between aluminum alloy wires in dry and lubrication conditions were evaluated. Additionally, under dry conditions, the percentage of aluminum adhesion was quantified using binarized SEM images processed with GIMP (v. 3.0.4).

In lubrication conditions, the lubricant was applied using a dropper, and the film thickness was not controlled. As a result, only a thin film of lubricant was applied, but the precise thickness was not ensured.

## 3. Results

### 3.1. Wear Mechanism Identifications During Sliding Contact with WC/Co Ball and Aluminium Wires Under Dry Condition

**Figure 2:**
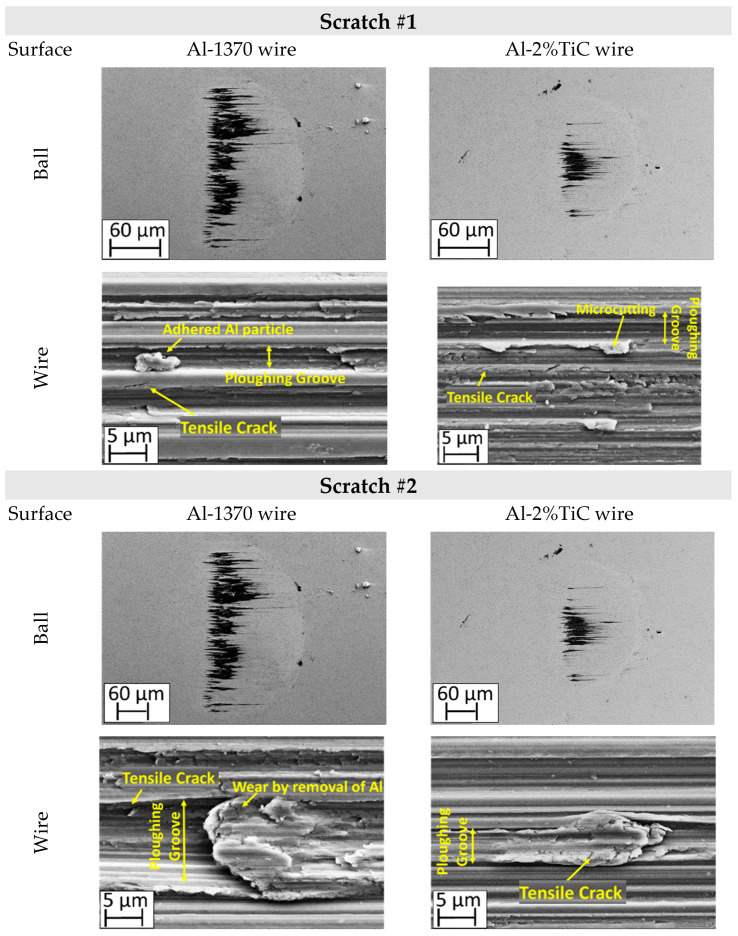

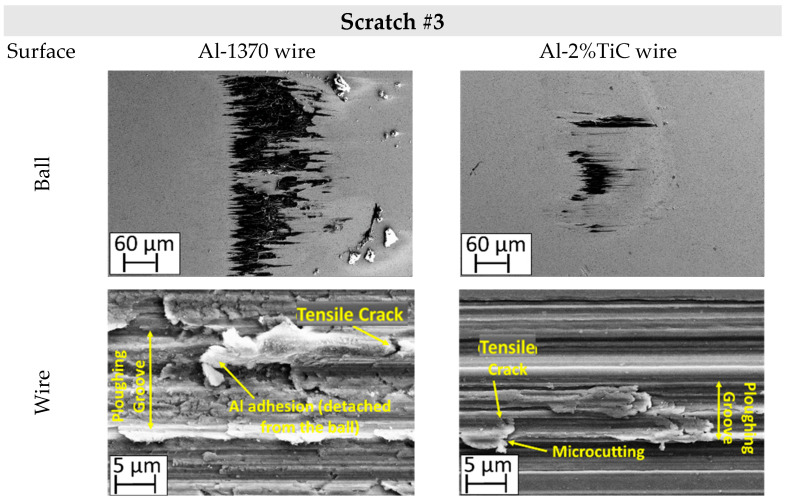
Ball/wire wear damage mechanisms evolution during the sliding contact between the WC/Co ball and the Al1370 (**left column**) wire and Al-2% TiC wire (**right column**).

**Figure 3:**
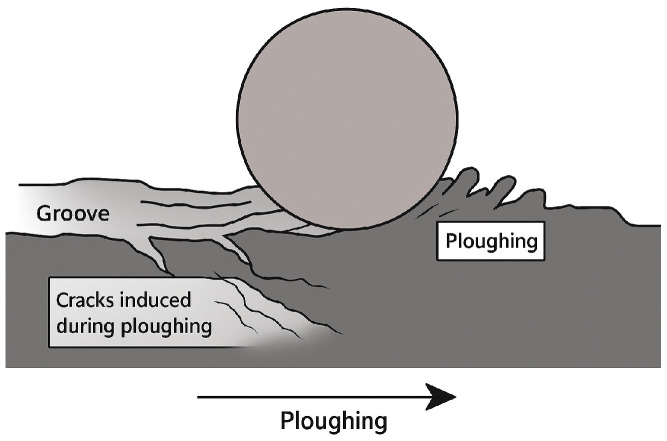
Schematic of the micro-ploughing wear mechanism observed on aluminum wires.

First, the modified scratch test was performed under dry condition and using a WC/Co ball, to identify the main damage mechanisms during the sliding contact against aluminium wires Al-1370 and Al-2% TiC. Figure 2 exhibits the identified wear mechanisms on both the aluminium wires and the WC/Co ball surface under dry conditions. The main wear mechanism observed in dry conditions is the aluminum material transfer, namely galling. It can be observed that during the sliding, the contact area between the WC/Co ball and the wire increases, significantly along the Al-1370 scratch tests. When the Al-1370 wire is drawn, adhered material is distributed in front of the ball, achieving up to 14.6% of the transferred aluminium in the semi-spherical contact zone after the third scratch (see Figure 3). In contrast, for Al-2%TiC, adhesion is concentrated in the central zone of the contact diameter, where the adhered aluminium covers only 2.7%. The adhesion increases in the absence of TiC nanoparticles; therefore, the friction between the hardmetal ball and the Al-1370 wire increases up 3 times compared with Al-2%TiC. Therefore, the presence of TiC particles reduces the adhesion wear mechanism due to its hardening effect.

On the other hand, one of the main wear damage mechanisms observed in aluminum wires is ploughing, i.e., the formation of grooves due to the different hardness values between the harder WC/Co ball and the softer wires (Figure 3). The asperities of the harder WC/Co ball penetrate the softer aluminum and, because of the sliding, grooves are formed. Another reason for ploughing, is the presence of hard worn particles present in the contact zone; this effect is evidenced by the presence of a microcutting wear mechanism at the edge of some grooves. Particle detachment from the WC/Co ball as a wear mechanism is discussed in detail in Section 4. In any case, ploughing produces high tensile stress in the worn surface (aluminum wires), leading to the formation of tensile cracks (Figure 3).

Figure 2 shows that ploughing appears on both wires’ materials, and tensile cracking occurs even in the first scratch test. In the case of the Al-1370 wire, the origin of the tensile cracks is mainly due to the large difference in hardness between the wire and the WC/Co ball in addition to the ductility of pure aluminum. Al-2%TiC has a higher hardness due to the TiC particle reinforcement, and grooves are narrower and shallower than in Al-1370. Nevertheless, tensile cracking also appears in the first step of the test, which is mainly due to the stress concentrating effect of the introduced TiC particles. Along the scratch tests, tensile cracks appear in the pile-up of the contact due to the slight deformation capacity of this material (*n* = 0.05 ± 0.01), and pile-up at the edge of the groove acts as a stress concentrator, which becomes the origin of cracking. In future work, additional cross-sectional analyses of the samples should be conducted to clearly identify the exact origin and propagation mechanism of these cracks.

The previous results indicate that the main wear mechanisms to be mitigated on the hardmetal surface are aluminum adhesion and ploughing, aimed at enhancing the wire surface quality.

### 3.2. Influence of Lubrication on Surface Quality of Aluminium Wire During WC/Co Ball Sliding Contact

In the previous section, ploughing was the main wear mechanism identified affecting surface wire quality. Due to the hardness mismatch between the WC/Co ball and the wires in combination with aluminium adhesion, ploughing takes place and, consequently, tensile cracks appear in the ploughing groove. Sliding entails shear stress increasing the probability of plastic flow and material fracturing in the contact region; hence, a well-accepted strategy to reduce shear stress is lubrication. Thus, a friction test was carried out following the experimental parameters described in Section 2.2 in lubricated conditions as this represents a common industrial strategy to diminish both adhesion and ploughing effect.

**Figure 4:**
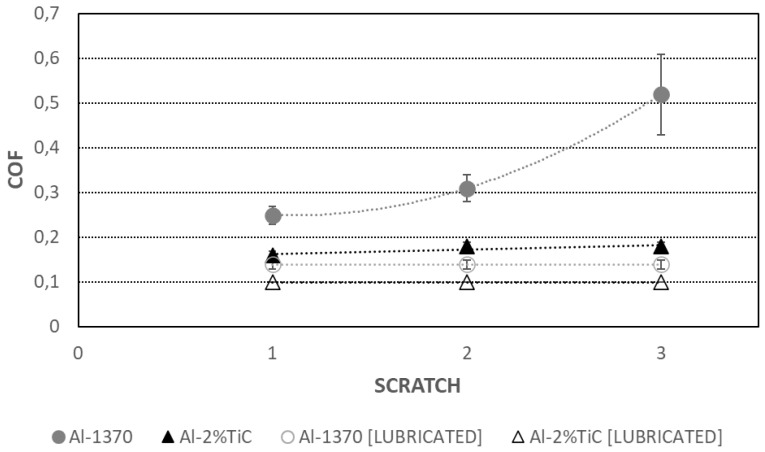
COF values obtained in dry (closed symbols) and lubricated conditions (open symbols) during the sliding of a WC/Co ball over Al-1370 (circles) and Al-2%TiC (triangles) wires.

**Figure 5:**
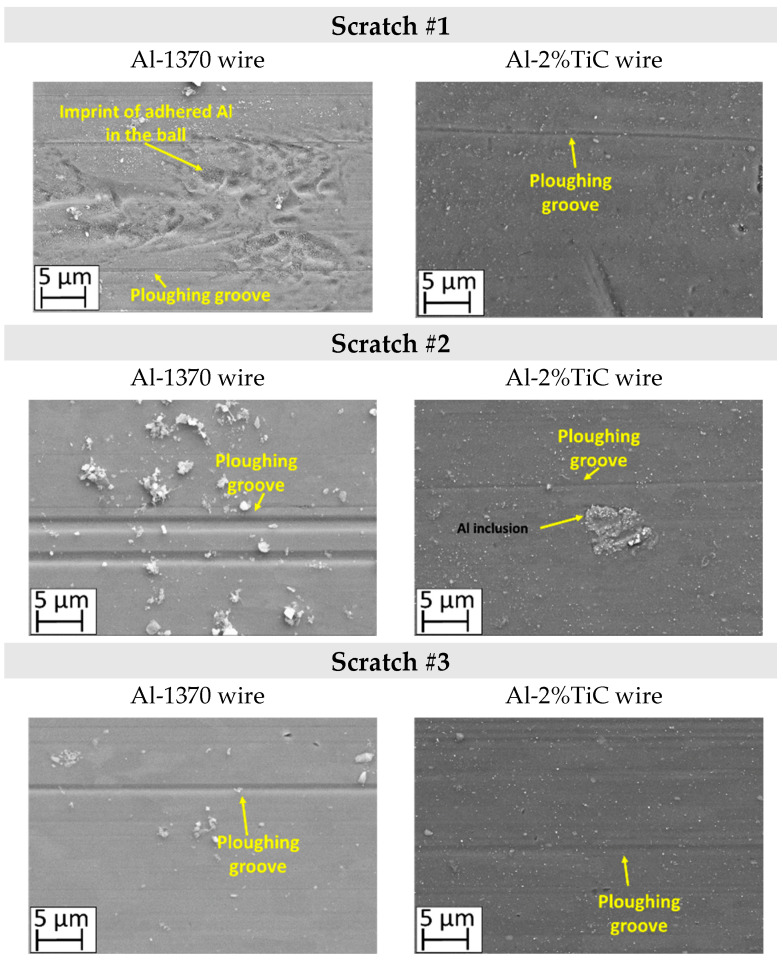
Wire wear damage mechanism evolution during the sliding contact between the WC/Co ball and the Al1370 wire (**left column**)and Al-2% TiC wire (**right column**) in lubricated conditions.

Figure 4 presents the COFs measured during the sliding of a WC/Co ball over aluminum wires in dry and lubricated conditions. The ball slid across the polished surfaces of 2 mm diameter Al-1370 and Al-2% TiC wires. The COF values are mean values along the scratch track, excluding the running-in period. Lubrication significantly reduces the COF. Due to the hardening effect of the TiC reinforcement particles, the COF is 3 times lower for the Al-2%TiC wire. The COF increases along the scratch test for the Al-1370 wire, confirming the galling observed in Figure 2. Furthermore, lubrication lowers the COF of the Al-1370 wire to levels below those measured for the Al-2%TiC wire under dry conditions, indicative of reduced or inhibited galling, as can be seen in Figure 5.

**Figure 6:**
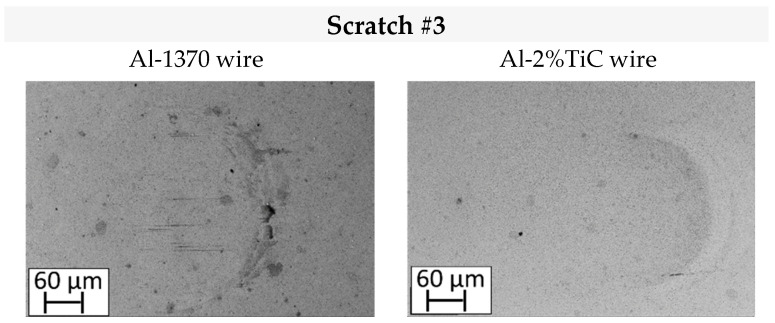
Counterbody (ball) surfaces after scratch #3 under lubrication.

Wear damage mechanisms in both the WC/Co balls and the aluminum wires were investigated to understand the COF behaviour. Figure 5 shows the identified wear mechanisms by means of SEM on the aluminum wires and the WC/Co ball surface under lubricated conditions. In the case of a WC/Co ball surface, wear mechanisms were examined only at the end of the test. After the third scratch, the ball was extracted and examined using SEM to ensure the preservation of the lubricant layer during the test. Figure 6 shows the identified wear mechanisms in the WC/Co ball.

Figure 5 does not show any tensile cracking, as they are inhibited by lubrication. Lubrication reduces the shear stresses in the contact zone, reducing both plastic flow and fracturing as tensile cracks. Nevertheless, adhesion is not eliminated in the case of Al-1370, which exhibit ploughing grooves. Adhesion is not observed at the end of the test when the WC/Co ball slides over the Al-2%TiC wire, but some shallow ploughing grooves can be observed. As mentioned in Section 3.1, ploughing can occur due to particle detachment from the WC/Co ball. In that sense, in scratch test #2 (Figure 5), an inclusion over the wire surface can be observed. The origin of this inclusion is probably due to drawn or pressed-in Al-adhered material on the WC/Co ball and transferred again into the wire surface. This wear mechanism is discussed further in Section 4. The film thickness of the lubricant was not controlled during the test; thus, the ploughing grooves in the Al-2%TiC wire could also be produced by the WC/Co ball and the punctual contact of the ball and the wire (i.e., boundary to mix lubrication).

In summary, the application of a lubricant between the WC/Co ball and the wire decrease significantly and even eliminate the quantity of aluminum adhered to the WC/Co ball surface. Consequently, marked scratches are drastically reduced. In this study, only a thin film of lubrication was applied; then, asperities from the WC/Co ball could exceed the lubrication film leading to scratches over the wire surface.

## 4. Discussion

Results indicate that adhesion and ploughing grooves are the primary damage mechanisms responsible for determining the final surface quality of WAAM aluminum wires. Two main origins of ploughing were detected: (1) aluminium adhesion and, (2) detachment of particles adhered in the WC/Co ball and transferred into the surface wire. In this section, the main damage micro-mechanisms that entail ploughing grooves in the wire surfaces are discussed. The inhibition of ploughing grooves will also entail the disappearance of tensile cracks due to the reduction or even the inhibition of the main wear mechanisms.

**Figure 7:**
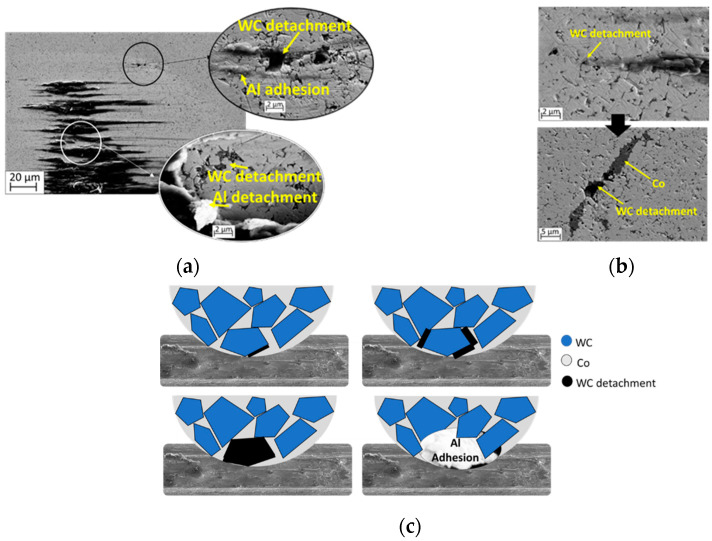
Observed damage micromechanisms in the WC/Co ball after the sliding test under (**a**) dry and (**b**) lubricated conditions. (**c**) Scheme of the WC particle detachment damage micromechanism in the WC/Co ball during sliding.

Figure 7 shows the main micromechanisms detected to be responsible for the groove formation, wich is the appearance of microcutting in the pile-up of the grooves as well as of aluminum adhesion. The main damage micromechanism is WC particles detachinh from the WC/Co ball. The soft binder phase (Co) in the cemented carbide exhibits preferential wear, leading to the selective detachment of the WC particles. Although scarcely documented in drawing contacts, some authors reported analogous pull-out during aluminum machining. Different authors have observed the presence of cavities on the WC/Co tool surface caused by WC dislodging during the machining of aluminum, attributed to the preferential wear of its soft binder (Co) [29,30,31,32,33]. However, even though this wear mechanism is also reported in other soft materials such as copper during continuous sliding contact with hardmetal tools [32], it is not well described during drawing processes [29,34]. This study supports that not only the bounded aluminium act as an abrasion particle, but the carbides of the WC/Co die play also an important role in the striation of the aluminium wire.

When Al or TiC abrasive particles are smaller or similar in size to tungsten carbide, they can interact separately with both the WC particles and the Co binder phase in the hardmetal [29,34]. Therefore, the binder phase is removed, and the carbide particle is pulled out of the WC/Co ball. Once the WC particle is detached from the surface, it is transferred to the wire surface, acting as a sharp abrasive particle inducing grooves and scratches over the drawn wire surface. At the same time, the hole left in the WC/Co ball after the WC particle detachment is filled with aluminum transferred from the wire, increasing the aluminum adhesion wear mechanism. Figure 7 illustrates the repeated sequence observed throughout the friction test: the amount of aluminium increase over the WC/Co ball surface until it is detached and transferred to the wire as a blunt particle, entailing adhesion, grooves, and plastic deformation.

Concerning ploughing grooves, two main different onsets were identified in Section 3: (1) WC particle detachment and (2) aluminum particle detachment. Both act as a third-body wear mechanism, since the particles (WC or Al) are transferred to the wires. During the sliding, these particles can roll and slide down the ball surface and scrape the soft wire surface, causing ploughing grooves. Ploughing produces high tensile stress in the worn surface, leading to the formation of tensile cracks. Consequently, inhibiting the ploughing effect will eliminate tensile cracking, improving the surface finish of the WAAM-produced wire. The results demonstrate that tensile cracks form both in the pile-up at the edge of the ploughing groove because of galling, and as a consequence of micro-cutting arising from the dislodgment of WC or adhered aluminum particles from the ball. The presence of hard TiC nanoparticles in the wire introduces new or exacerbates existing wear mechanisms on the die. For instance, the presence of secondary phases can facilitate the spalling of the free-moving solid aluminum particles. These debris roll and slide during the contact between the tool and the wire, leading to third-body abrasion [35]. The third-body wear mechanism directly impacts COF. During the initial steps of sliding, the COF decreases when a transfer film forms between the tool and the wire; it is expected to increase if interface debris accumulates and deforms the wear track [35]. Therefore, third-body abrasion caused by aluminum adhesion and by the presence of free dislodged WC particles represents a major concern for maintaining wire surface quality. Third-body abrasion promotes the accumulation of impurities on the surface [35] and contributes to fatigue failure [36]. In addition, tool life is also reduced because the detachment of WC carbides weakens the hardmetal matrix [35]. The present study demonstrates that the use of nanoparticle-reinforced aluminium is an effective industrial strategy to mitigate third-body abrasion mechanisms. Lubrication further decreases shear stresses, helping to prevent adhesion. However, the size of the nanoparticles plays a critical role in the onset of WC particle detachment. Although lubrication that forms a transfer film between the WC/Co and the wire reduces the WC detachment, it does not eliminate it entirely. Consequently, further research is required to improve the interaction between the hardmetal die and the TiC-reinforced aluminium wire.

This study represents an initial stage; future work should focus on optimizing the nature of lubricants and the application conditions during wire production. Further effort is also needed to evaluate combinations of lubrication with the application of thin low friction surface coatings on the hardmetal die. Such coatings are expected to inhibit WC particles detachment and thereby improve the final surface quality of the wire. In this context, selecting an appropriate coating is critical. Surface roughness, a reduction in microdroplets, and elimination of growing disrupts in the coatingsare essential factors for reducing friction, limiting aluminum adhesion, and protecting the WC-Co substrate.

## 5. Conclusions

The findings obtained in this study provide new insights into the wear mechanisms governing the interaction between TiC- reinforced aluminium wires and WC/Co die. The following conclusions summarize the most relevant contributions of this study and their implications for improving process reliability, tool life, and final wire surface quality:

- Under dry conditions, the transferred material from wire to the ball was significantly reduced from 14.7% to 2.7%, when TiC- reinforced aluminium wire is used. Therefore, the friction between the hardmetal ball and Al-2%TiC wire also has de-creased up 3 times compared with the Al-1370. This is clear evidence that the presence of TiC particles reduces the adhesion wear mechanism due to its hardening effect.
- Not only the bounded aluminium (galling) act as an abrasion particle, but the carbides of the hardmetal paly also an important role in the surface quality of the aluminum wire. Wear debris produced scratch on both wires surface, however deeper tracks were discerned on Al-1370 wires.
- The reduction of ploughing effect leads to improved surface finishing of WAAM-produced wires. This can be achieved by introducing TiC particles and by using lubrication, as these methods are complementary. Therefore, their combined use is recommended: TiC particles reduce surface defects, while lubrication reduces the shear stresses that contribute to formation ploughing grooves formation.
- For future work, the following guidelines and recommendations should be addressed to improve the surface quality of particle-reinforced aluminium WAAM during the drawing process with WC/Co dies:
  (1) Hardness: reducing the hardness mismatch between the aluminium matrix and the reinforcement particles will reduce the incidence of tensile cracks since the pressures during the drawing process are well distributed in the whole wire avoiding the stress concentrator effect of the reinforcement particles.
  (2) Lubrication: the use of lubricants reduces shear stress and, consequently, mitigates the observed wear mechanisms. Factors such as the lubricant’s composition and the specific application conditions during wire production must be carefully considered.
  (3) Nanoparticle size: The size of the reinforcement nanoparticles must be carefully selected, as it critically influences the onset of WC particle detachment. This wear mechanism can be mitigated by choosing an appropriate coating. Key factors for reducing friction, limiting aluminum adhesion, and protecting the WC-Co substrate to extend die life include controlling surface roughness, minimizing microdroplets, and eliminating growth disruptions in the coatings.

The main contribution of the paper is the mechanistic understanding of how reinforcement nanoparticles interact with WC/Co under sliding conditions, and how this interaction influences damage development at the contact interface.

## Figures and Tables

**Table 1:** Al-1370 and Al-2%TiC wires mechanical properties [12].

Specimen Ø 2 mm	R_p_0.2 [MPa]	R_p_0 [MPa]	*n*	HV0.5
Al 1370	34 ± 12	102 ± 0	0.32 ± 0.13	31 ± 2
Al-2%TiC	97 ± 16	129 ± 2	0.05 ± 0.01	44 ± 2

**Table 2:** WC/Co ball characteristics [12].

Diameter [mm]	Co [wt.%]	Grain Size [17,18]	Ra [µm]	H [GPa]
10	5	Submicron	0.23 ± 0.003	20.3 ± 0.6

## Data Availability

The original contributions presented in this study are included in the article. Further inquiries can be directed to the corresponding author.
